# Deep-Learning-Based Approach in Cancer-Region Assessment from HER2-SISH Breast Histopathology Whole Slide Images

**DOI:** 10.3390/cancers16223794

**Published:** 2024-11-11

**Authors:** Zaka Ur Rehman, Mohammad Faizal Ahmad Fauzi, Wan Siti Halimatul Munirah Wan Ahmad, Fazly Salleh Abas, Phaik-Leng Cheah, Seow-Fan Chiew, Lai-Meng Looi

**Affiliations:** 1 Faculty of Engineering, Multimedia University, Cyberjaya 63100, Malaysia; 1211400112@student.mmu.edu.my (Z.U.R.); halimatulmunirah@imu.edu.my (W.S.H.M.W.A.); 2 Institute for Research, Development and Innovation, IMU University, Bukit Jalil, Kuala Lumpur 57000, Malaysia; 3 Faculty of Engineering and Technology, Multimedia University, Bukit Beruang, Melaka 75450, Malaysia; fazly.salleh.abas@mmu.edu.my; 4 Department of Pathology, University Malaya-Medical Center, Kuala Lumpur 50603, Malaysia; cheahpl@ummc.edu.my (P.-L.C.); sfchiew@ummc.edu.my (S.-F.C.); looilm@ummc.edu.my (L.-M.L.)

**Keywords:** deep learning, digital pathology, human epidermal growth factor receptor 2 (HER2), silver-enhanced in situ hybridization (SISH)

## Abstract

Fluorescence in situ hybridization (FISH) is widely regarded as the gold standard for evaluating human epidermal growth factor receptor 2 (HER2) status in breast cancer; however, it poses challenges such as the need for specialized training and issues related to signal degradation from dye quenching. Silver-enhanced in situ hybridization (SISH) serves as an automated alternative, employing permanent staining suitable for bright-field microscopy. Determining HER2 status involves distinguishing between “Amplified” and “Non-Amplified” regions by assessing HER2 and centromere 17 (CEN17) signals in SISH-stained slides. This study is the first to leverage deep learning for classifying Normal, Amplified, and Non-Amplified regions within HER2-SISH whole slide images (WSIs), which are notably more complex to analyze compared to hematoxylin and eosin (H&E)-stained slides. Our proposed approach consists of a two-stage process: first, we evaluate deep-learning models on annotated image regions, and then we apply the most effective model to WSIs for regional identification and localization. Subsequently, pseudo-color maps representing each class are overlaid, and the WSIs are reconstructed with these mapped regions. Using a private dataset of HER2-SISH breast cancer slides digitized at 40× magnification, we achieved a patch-level classification accuracy of 99.9% and a generalization accuracy of 78.8% by applying transfer learning with a Vision Transformer (ViT) model. The robustness of the model was further evaluated through k-fold cross-validation, yielding an average performance accuracy of 98%, with metrics reported alongside 95% confidence intervals to ensure statistical reliability. This method shows significant promise for clinical applications, particularly in assessing HER2 expression status in HER2-SISH histopathology images. It provides an automated solution that can aid pathologists in efficiently identifying HER2-amplified regions, thus enhancing diagnostic outcomes for breast cancer treatment.

## Simple Summary

Accurate detection of HER2 status in breast cancer is vital for effective diagnosis and treatment. While traditional fluorescence in situ hybridization (FISH) is the standard for assessing HER2, it has limitations, such as the need for expert handling and potential signal fading. Silver-enhanced in situ hybridization (SISH) offers a more stable alternative for analysis using conventional bright-field microscopy. This study explores an innovative deep-learning approach to automatically classify Normal, Amplified, and Non-Amplified regions in HER2-SISH images. Our two-stage process trains and evaluates models on annotated patches and applies the most effective model to entire slide images, generating region-specific visualizations. This method not only reduces manual workload but also shows strong potential for aiding pathologists in identifying HER2-amplified areas, thereby supporting precise breast cancer treatment. Statistical evaluations, including k-fold cross-validation and confidence intervals, further validate the robustness of our approach.

## 1. Introduction

Breast cancer arises from the uncontrolled growth of cells in the body, with an estimated 10–20% of initial cases involving mutations in the HER2 protein. Before the advent of HER2-targeted therapies, these mutations were often associated with an aggressive phenotype and poor clinical outcomes [1]. Patients diagnosed with HER2-positive malignancies now have access to targeted treatments, including trastuzumab (Herceptin), lapatinib (Tykerb), pertuzumab (Perjeta), and ado-trastuzumab emtansine (T-DM1, Kadcyla). While these therapies have greatly improved prognosis, they come with potential side effects and high costs, making precise identification of HER2-amplified tumors crucial for accurate diagnosis and effective treatment [2,3]. Consequently, accurate detection of HER2 amplification remains an essential part of breast cancer management [4].

Various diagnostic methods are used to evaluate HER2 status at the genetic level, including fluorescence in situ hybridization (FISH), chromogenic in situ hybridization (CISH), reverse transcription-polymerase chain reaction (RT-PCR), and immunohistochemistry (IHC). Among these, FISH has been widely adopted for its reliability but presents certain limitations, such as the need for specialized training, the use of fluorescence microscopy, and potential signal degradation due to dye quenching. Silver-enhanced in situ hybridization (SISH) offers a fully automated alternative that produces permanently stained slides suitable for interpretation with standard bright-field microscopy. This method allows pathologists to assess HER2 expression within the context of tissue morphology, providing a more stable and accessible means of evaluating HER2 status.

Despite the advantages of SISH, current methods largely rely on manual analysis by pathologists or traditional image analysis techniques that can be subjective and prone to variability. Although some automated approaches exist for evaluating HER2 scores using SISH, they often adhere strictly to clinical scoring methods without leveraging the full potential of modern computational techniques [5]. To date, there has been no research that employs deep-learning models to directly classify and identify amplified and non-amplified regions within HER2-SISH WSIs. The lack of automated systems capable of handling the complexity of SISH-stained slides highlights the need for advanced methodologies that can improve consistency, efficiency, and accuracy in HER2 scoring.

The digitization of entire slides into whole slide images (WSIs) has become feasible, allowing for comprehensive analysis [6]. However, manual review of histopathological WSIs, whether under a microscope or on a computer, can be labor-intensive, time-consuming, and prone to errors. Furthermore, diagnostic concordance among pathologists is estimated to be around 75%, largely due to the subjective nature of the morphological criteria used in image classification [7].

**Figure 1:**
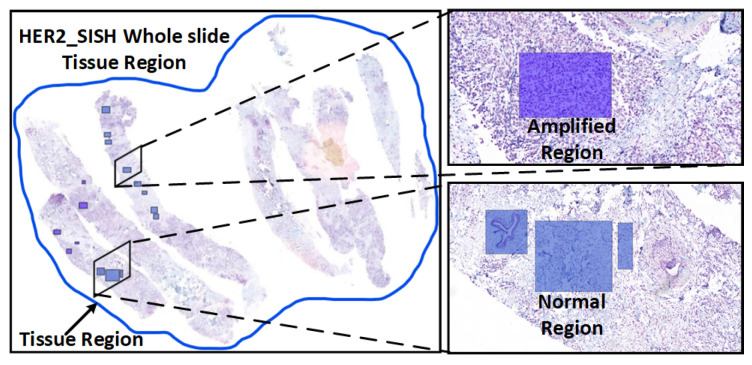
A Whole Slide Image (WSI) depicting tissue regions (**left**) and a magnified selected region (**right**) for detailed analysis of tissue anatomy, with Amplified and Normal regions marked by a pathologist for diagnostic purposes.

As shown in Figure 1, efforts to enhance diagnostic accuracy and consistency have led to the development of computer-aided diagnostic (CAD) systems that incorporate morphological criteria into traditional classification approaches [8]. Building a reliable CAD system for cancer classification based on histopathology images is challenging due to the inherent complexity of cancer. Fortunately, recent advances in machine learning, particularly deep learning, have greatly improved the feasibility and reliability of automated image analysis. Convolutional neural networks (CNNs), a popular deep-learning architecture, have demonstrated significant promise in classifying cancer histology images [9]. CNNs can automatically extract intermediate and high-level features from RGB images, making them effective for tasks such as object recognition, image segmentation, and target localization [10]. They have become the preferred approach for analyzing histopathological images and have shown strong performance in binary classification tasks, such as distinguishing between benign and malignant tumors, especially when combined with multiple instance-learning techniques [11].

However, automated analysis faces challenges related to color variations in histopathological images. Factors such as staining technique variations, differences in stain reactivity, scanner settings, and slide thickness can introduce color inconsistencies. While pathologists can often adapt to these variations, they can significantly impact computer-based image processing. To address these issues, stain normalization techniques have been developed, which help standardize images and enhance the robustness of automated analysis [12].

### Contribution of This Work

In this paper, we present a computer-aided system for the automatic identification of three region types—Normal, Amplified, and Non-Amplified—in HER2-SISH-stained WSIs and image patches. Our contributions include the following:

1. The preparation of a three-class image dataset by patchifying regions of interest (ROIs) identified by expert pathologists. The large ROIs were divided into patches of size (512×512×3), with outliers removed during the process.
2. The fine-tuning and evaluation of various transfer learning models on the HER2-SISH dataset.
3. The development of an automated system that segments WSIs into patches; classifies them as Normal, Amplified, or Non-Amplified; and overlays a pseudo-color map based on the model predictions. The system reconstructs the entire WSI using these predictions, eliminating manual steps and enhancing the accuracy and reliability of region identification.

The remainder of this paper is structured as follows: Section 2 reviews related work, while Section 3 outlines the methodologies and datasets used, including data preprocessing and the deep-learning models employed. Section 4 details the hardware specifications, training, and testing parameters, as well as the evaluation metrics. The results and analysis are presented in Section 5, followed by conclusions and future work in Section 6.

## 2. Literature Review

Artificial intelligence (AI) has played a crucial role in enhancing productivity and efficiency across various domains by enabling data-driven decision-making [13]. The combination of high-performance computing (HPC) [14], large datasets, sophisticated algorithms, and intensive research efforts has driven the widespread adoption of AI over the past decade. Beyond healthcare, AI techniques are employed in diverse fields such as network intrusion detection [15] and speech-based person identification [16]. In healthcare, AI has increasingly been used for diagnostic tasks, including the identification of pneumonia [17], diabetic retinopathy diagnosis, and glaucoma detection [18]. The field of digital histopathology, in particular, has witnessed a surge in the development of computer-aided diagnostic (CAD) systems aimed at augmenting or even replacing the optical microscope as a primary tool for pathologists [19].

CAD methods are broadly categorized into two types: (1) deep-learning algorithms that process raw data to automatically extract features and support data classification, and (2) traditional machine learning algorithms that use handcrafted features extracted from data [20]. Early success in CAD systems was primarily driven by standard machine-learning techniques that relied on domain expertise to manually extract features for classification tasks.

In the initial phases of histopathology image classification, machine-learning techniques such as texture descriptors were widely used for feature extraction. Techniques like the Gray Level Co-occurrence Matrix (GLCM), Pyramid-Structured Wavelet Transform (PWT), Local Binary Patterns (LBP), and Tree Structure Wavelet Transform (TWT) were employed for feature extraction and subsequent classification. For instance, Samah et al. [21] utilized features derived from GLCM, PWT, LBP, and TWT to distinguish between benign and malignant tumors, using a K-Nearest Neighbor (KNN) classifier. This approach demonstrated improved classification performance, particularly when using GLCM features.

Spanhol et al. [22] also employed feature extraction methods such as Local Phase Quantization (LPQ) and LBP for the binary classification of breast cancer histopathological images. Various features, including GLCM, Oriented FAST and Rotated BRIEF (ORB), and Threshold Adjacency Statistics (TAS), were combined with classifiers such as 1-NN, Quadratic Discriminant Analysis (QDA), Random Forest (RF), and Support Vector Machine (SVM), achieving accuracies ranging from 80 to 85%.

Ojansivu et al. [23] developed a texture-based approach using an SVM classifier for the automatic classification of breast cancer morphology, while Weyn et al. [24] applied wavelet-derived textural features to differentiate between high- and low-grade tumor nuclei in breast tissue. Doyle et al. [25] combined textural and architectural features to classify low- and high-grade Nottingham tumors. However, most of these methods either focused on a single aspect of the Nottingham grading system or had limited datasets. Basavanhally et al. [26] expanded on this by proposing a multi-field-of-view framework that integrated textural and graph-based features for grading estrogen receptor-positive breast cancer histopathology WSIs. These advancements underscore the growing demand for automated image analysis techniques that incorporate multilevel features for accurate tumor classification.

In the context of deep learning (DL), researchers have increasingly adopted advanced techniques for histopathology image analysis. Spanhol et al. [27] used the BreakHis dataset to develop a deep-learning model with AlexNet for the binary classification of breast cancer histopathological images, achieving the highest accuracy at 40× magnification. Han et al. [28] explored multiclass classification through a CNN-based approach utilizing hierarchical feature representation, demonstrating high reliability in breast cancer classification. For the BACH dataset, Roy et al. [29] proposed a patch-based classifier using a CNN and two strategies: One Patch in One Decision (OPOD) and All Patches in One Decision (APOD). OPOD classified each patch independently, while APOD assigned an image-level label based on majority voting across patches.

Beyond classification, CNNs have proven effective for learning discriminative features in histopathology images. Ciresan et al. [30] adapted a deep max-pooling CNN for mitosis detection in breast histology images, treating detection as a pixel-level classification task and achieving first place in the ICPR 2012 mitotic detection competition. Cruz-Roa et al. [31] developed a deep-learning model for basal cell carcinoma detection, training on a set of 1417 images from 308 regions of interest in skin histology slides. Transfer learning from the ImageNet database [32], with its 14 million images, has become a widely adopted practice, serving as a strong foundation for CNN models applied to histopathology tasks.

The role of deep learning in enhancing histopathological image analysis is underscored by studies such as those presented by Cha et al. [33], who demonstrated the potential of machine-learning models in classifying tumor types and predicting patient outcomes, and Ahmed et al. [34], who demonstrated the use of machine learning in biomedical image processing for extracting complex patterns. Additionally, the application of CNNs in medical imaging has proven effective for integrating multilevel features, enhancing both the accuracy and reliability of tumor classification [35,36].

Fine-tuning pre-trained CNNs has emerged as an effective approach for enhancing classification performance in specialized applications. Girshick [37] demonstrated substantial improvements in object detection through fine-tuning, while Zhang et al. [38] reported significant gains in fine-grained classification tasks after fine-tuning pre-trained CNN models. In this study, we employ fine-tuning to optimize CNN models for histopathological image classification, leveraging pre-trained ImageNet models as a starting point.

Despite the extensive research on deep-learning applications in histopathology, the classification of HER2-SISH WSIs into Normal, Amplified, and Non-Amplified regions has not been thoroughly explored. To the best of our knowledge, no existing methods address the classification of HER2-SISH images into three distinct classes or the identification of regions within WSIs for this specific stain. This study aims to fill this gap by proposing a novel deep-learning-based approach for HER2-SISH image classification and WSI region identification.

## 3. Material and Methodology

**Figure 2:**
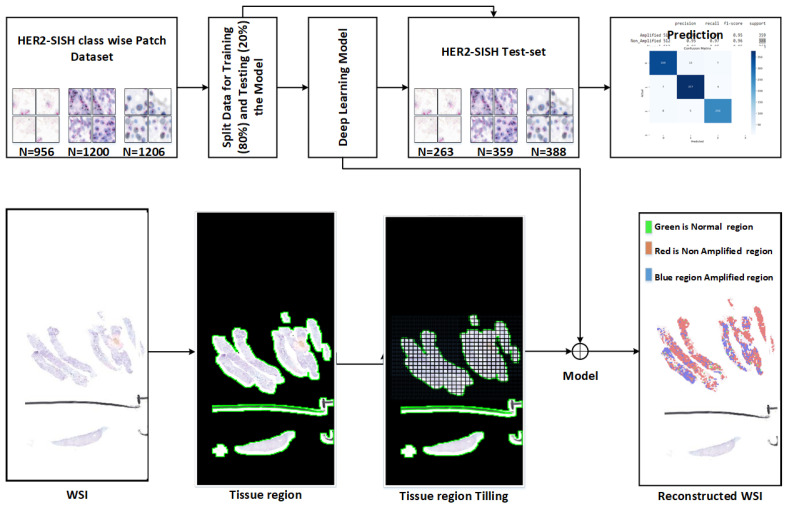
Proposed framework for patch-based image classification and identification of respective class samples from the whole slide image (WSI) using the trained model.

**Figure 3:**
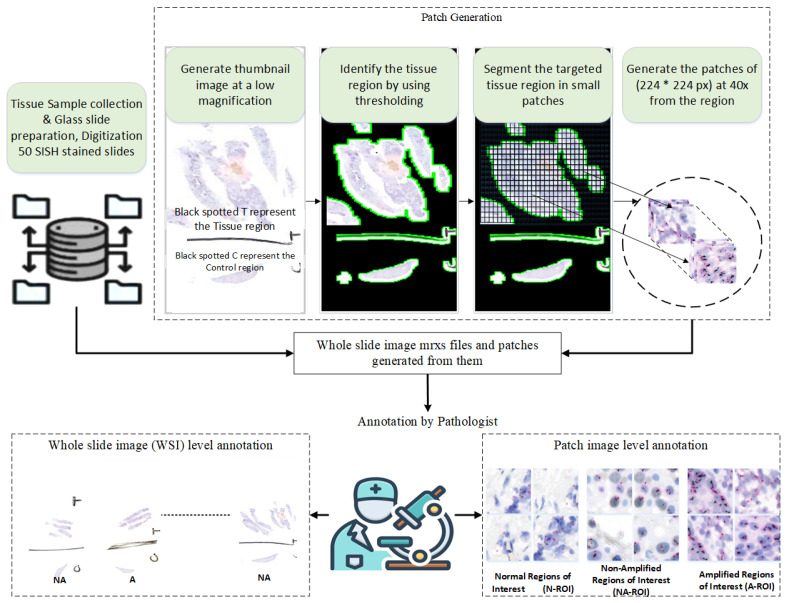
Procedural diagram illustrating the automated selection of tissue regions and image patching from whole slide images (WSIs), with expert-level annotation.

This section details the methodologies employed in the proposed approach, encompassing the dataset, model architecture, and evaluation of generalization performance on unseen data. Figure 2 and Figure 3 provide an architectural overview of the proposed workflow.

### 3.1. Dataset Description

**Figure 4:**
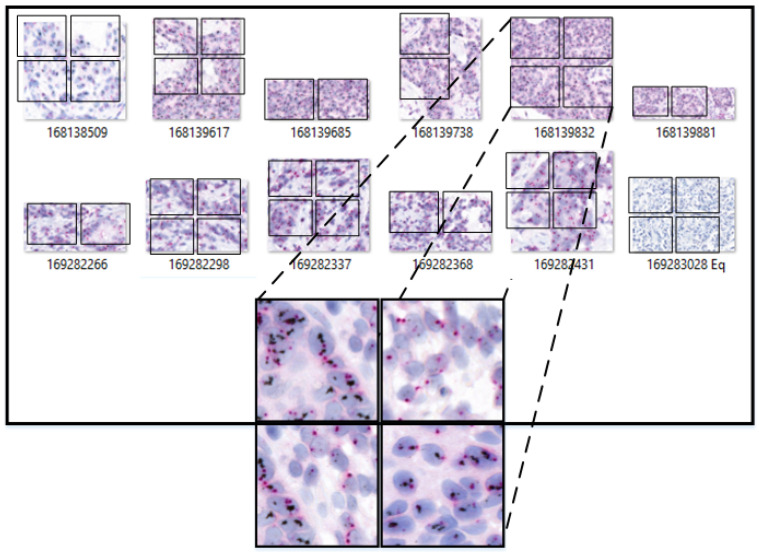
Annotated regions in the images have irregular shapes. A segmentation process standardizes their shape to 512×512×3 pixels by sliding a window from the top-left corner horizontally and vertically to cover the entire region of interest. Portions outside the window are discarded.

The clinical dataset for this study was obtained from our collaborating hospital, with the following details:

1. The HER2-SISH dataset includes annotated Normal, Amplified, and Non-Amplified regions within whole slide images (WSId. To our knowledge, this is the first HER2-SISH WSI dataset providing image-based annotations for these three region types.
2. Tissue samples from 50 patients were collected and stained with silver-enhanced in situ hybridization (SISH) for HER2. The slides were scanned using the 3DHistech Pannoramic DESK, resulting in 50 WSIs at 40× magnification with dimensions of approximately 250,368×572,416 pixels. Of these, 46 WSIs met the quality criteria for inclusion, while four were excluded due to issues such as low resolution, staining artifacts, or physical damage. Expert pathologists annotated regions of interest (ROIs) for HER2 Normal, Amplified, and Non-Amplified areas, saved as 8-bit/channel RGB TIFF images. Figure 4 shows examples of ROIs selected by pathologists from the WSIs. Table 1 summarizes the statistical details of the WSIs and ROIs. Additionally, Dataset 2, consisting of 9 WSIs, was used to test the model’s generalization on unseen data.

### 3.2. Preprocessing

Histopathological images are inherently complex and large, presenting significant challenges for machine-learning algorithms due to their detailed texture and high resolution [39]. These high-resolution images offer crucial diagnostic information, which advanced image analysis techniques aim to utilize for supporting expert decision-making, facilitating consensus, saving time, and detecting visual patterns that may otherwise be overlooked [40].

In this study, we propose an automated deep-learning method that identifies and classifies HER2 Amplified, Non-amplified, and Normal regions from WSIs. The annotated regions of interest (ROIs) were provided by expert pathologists and consisted of large, irregularly shaped regions that varied significantly in size. Resizing these large ROIs directly would result in a loss of important image details and texture, thus compromising the model’s performance.

**Figure 5:**
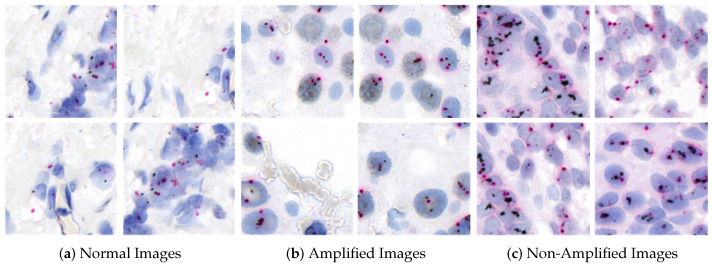
Examples of HER2-SISH patch samples categorized into their respective classes: (**a**) Normal, (**b**) Amplified, and (**c**) Non-Amplified.

To address this challenge, we employed image-patching, a technique that divides large ROIs into smaller, more manageable segments. Specifically, we used non-overlapping patches of size 512×512×3 pixels by convolving a window of the same size with a stride equal to the patch size to ensure that the patches did not overlap. This approach preserved the details and texture within each patch while maintaining the original magnification levels, as shown in Figure 5.

To standardize the images for model training, the 512×512 patches were resized to 224×224 pixels, making them compatible with standard deep-learning architectures. This resizing was carefully executed to ensure that no critical features were excluded, thus preserving the diagnostic integrity of the images.

Additionally, data augmentation techniques, such as rotation, flipping, and scaling, were applied during model training to increase the variability of the training dataset and improve the model’s generalization ability.

The impact of these preprocessing steps was significant, as the image-patching method allowed the model to learn from detailed, consistent segments without losing essential information. This approach improved the training process by ensuring that each patch contained sufficient information for classification, ultimately contributing to the robustness and accuracy of the model’s performance.

### 3.3. Deep-Learning Models

This study evaluated four deep-learning models: DenseNet121, VGG16, MobileNetV2, and Vision Transformer (ViT). Each model’s performance was assessed in classifying HER2-SISH regions as Normal, Amplified, or Non-Amplified.

The ViT model exhibited superior performance, achieving the highest accuracy in both patch-level and WSI-level evaluations. While detailed descriptions of VGG16 and MobileNetV2 architectures are omitted for brevity, their structures are well-documented in the literature [41,42].

We selected four widely adopted architectures: DenseNet121, VGG16, MobileNetV2, and Vision Transformer (ViT), each chosen for its strengths in image classification tasks, particularly in medical image analysis. Detailed descriptions of these architectures and their contributions are provided below.

#### 3.3.1. DenseNet121 Architecture

**Figure 6:**
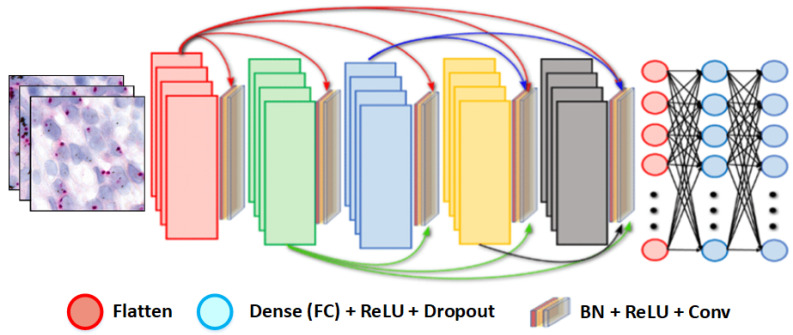
Overview of the DenseNet121 architecture.

DenseNet121 is a convolutional neural network architecture comprising multiple dense blocks [43,44]. Its innovation lies in dense connections between layers, where each layer receives input from all preceding layers within the same block, facilitating feature reuse and mitigating the vanishing gradient problem. The network has 121 layers and approximately 20 million parameters. Figure 6 depicts the architecture of DenseNet121.

For this study, a pre-trained DenseNet121 model initialized with ImageNet weights (from 1.2 million images across 1000 categories) was fine-tuned. The original output layer was replaced with a three-neuron layer corresponding to the HER2-SISH classes (Normal, Amplified, Non-Amplified), with a softmax activation function for probability output.

#### 3.3.2. VGG16 Architecture

VGG16, introduced by Simonyan and Zisserman in their work “Very Deep Convolutional Networks for Large Scale Image Recognition” [41], is designed for high performance in image classification. It operates on 224×224×3 input images, passing them through convolutional and max-pooling layers.

**Figure 7:**
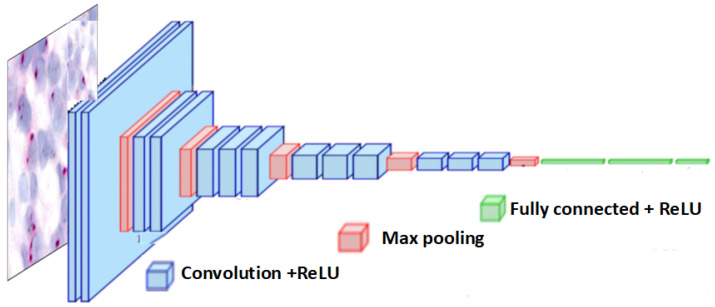
Overview of the VGG16 architecture.

The architecture includes 16 weight layers, 13 convolutional and 3 fully connected layers, with filter counts increasing from 64 to 512. ReLU activation follows each convolution. The VGG16 architecture is effective for feature extraction and classification, as illustrated in Figure 7.

#### 3.3.3. MobileNetV2 Architecture

MobileNetV2 is an efficient convolutional neural network for mobile and embedded devices [42], featuring inverted residual blocks and depth-wise separable convolutions to minimize computational cost while maintaining accuracy.

**Figure 8:**
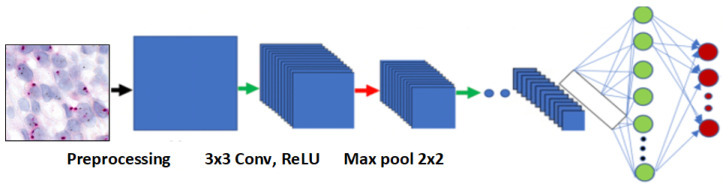
Overview of the MobileNetV2 architecture.

The model uses bottleneck layers to reduce input and output channels, enhancing efficiency. Linear bottlenecks increase representational capacity without added complexity, and shortcut connections aid gradient flow. Figure 8 provides an overview of the MobileNetV2 architecture.

#### 3.3.4. Vision Transformer (ViT) Architecture

The Vision Transformer (ViT) architecture, based on the original Transformer model used in natural language processing [45], has achieved notable success in image classification. Unlike traditional CNNs, which process the image as a whole, ViT divides input images into patches and treats each patch as a token, akin to word processing in NLP.

**Figure 9:**
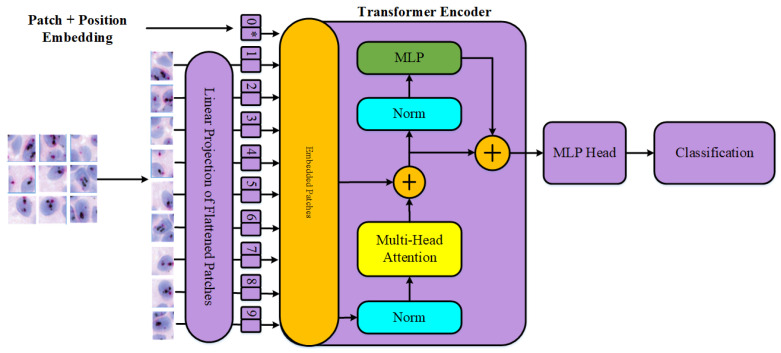
Overview of the Vision Transformer (ViT) architecture: The model divides input images into patches and treats each patch as a token for processing.

This patch-based approach enables ViT to capture long-range dependencies within an image, making it effective for classification tasks. The self-attention layers provide a global understanding of the image. Figure 9 depicts the Vision Transformer model’s structure.

### 3.4. Hyperparameters

Optimal hyperparameters are crucial for model performance. Table 2 presents the hyperparameters used during training and fine-tuning for DenseNet121, VGG16, MobileNetV2, and Vision Transformer (ViT) models. These parameters were selected through iterative experimentation and prior research to optimize the classification of HER2-SISH regions.

## 4. Experimental Setup

This section outlines the experimental setup utilized to evaluate the performance of the proposed models in classifying Normal, Amplified, and Non-Amplified regions within HER2-SISH WSIs. It includes descriptions of the hardware used, training and testing methodologies, and the evaluation metrics applied to assess model effectiveness.

### 4.1. Hardware Specifications

The proposed HER2-SISH histopathology image classification system was implemented and tested on the following hardware configuration:

- Operating System (OS): Windows 11 (64-bit)
- Intel CPU: Intel Core i7 @ 2.40 GHz
- Sammsung DDR4 RAM: 32 GB
- Nvidia GTX4060 GPU: 8 GB Graphics Card

The development environment was based on Python 3.9, with CUDA 11.2, cuDNN 8.2, and OpenCV 3.0. The DeepZoom generator function [46] was employed to manage WSIs for this experiment.

### 4.2. Training and Testing Methodology

The initial phase of the methodology involved preprocessing data and annotations sourced from medical institutions to prepare them for model training. Non-overlapping regions of interest (ROIs) were divided into patches of size 512×512×3, with 80% used for training (leveraging pre-trained ImageNet weights) and 20% set aside for testing.

The dataset was split in an 80–20 ratio for training and testing on Dataset 1, ensuring that patches from the same WSI did not appear in both sets to prevent data leakage. The term test results refers to performance on the dedicated 20% testing subset, while generalization performance pertains to the model’s performance on an independent dataset (Dataset 2) comprising 9 WSIs not included in the training phase.

Given the relatively small dataset, leave-one-out cross-validation (LOOCV) was also explored to assess the model’s generalization capacity more accurately and mitigate dataset size constraints.

Standard image augmentation techniques were employed to enhance the training set’s diversity. The hyperparameters used for MobileNetV2, VGG16, and DenseNet121 are presented in Table 2, with variations in the ViT model’s hyperparameters due to its unique architecture.

Lastly, it should be noted that applying our approach to publicly available histopathology image datasets was not feasible due to the absence of datasets containing three-class annotations and corresponding WSI images.

### 4.3. Evaluation Metrics

Evaluating model performance in medical image classification extends beyond accuracy, as misclassifications can have significant implications. Therefore, we employ a range of metrics such as F1-score, sensitivity (recall), specificity, precision, and additional comprehensive metrics to provide a thorough assessment. These metrics facilitate the evaluation of model performance for each class independently, regardless of the dataset’s class distribution. The following equations define these metrics:

(1) $$\mathrm{Accuracy}=\frac{TP+TN}{TP+TN+FP+FN}$$

(2) $$\mathrm{Sensitivity}\;\left(\mathrm{Recall}\right)=\frac{TP}{TP+FN}$$

(3) $$\mathrm{Precision}=\frac{TP}{TP+FP}$$

(4) $$F1-\mathrm{score}=\frac{2\times (\mathrm{Sensitivity}\times \mathrm{Precision})}{\mathrm{Sensitivity}+\mathrm{Precision}}$$

(5) $$\mathrm{Balanced}\;\mathrm{Accuracy}=\frac{1}{2}\left(\frac{TP}{TP+FN}+\frac{TN}{TN+FP}\right)$$

(6) $$\mathrm{MCC}=\frac{TP\times TN-FP\times FN}{\sqrt{\left(TP+FP)(TP+FN)(TN+FP)(TN+FN\right)}}$$

We also report the Area Under the Receiver Operating Characteristic Curve (AUC-ROC) and Cohen’s Kappa to evaluate the model’s discriminatory power and agreement with the true labels.

In this study, which focuses on classifying Normal, Amplified, and Non-Amplified regions in HER2-SISH histopathology images, the terms TP (True Positive), TN (True Negative), FP (False Positive), and FN (False Negative) are defined as follows:

- True Positive (TP): Instances where images correctly belong to a specific class (e.g., Normal).
- True Negative (TN): Instances where images correctly do not belong to a specific class.
- False Positive (FP): Instances where images are incorrectly classified as belonging to a specific class.
- False Negative (FN): Instances where images belonging to a specific class are incorrectly classified as not belonging to that class.

## 5. Results and Discussion

This section presents the findings from the implementation of the proposed methodology on the HER2-SISH datasets. We compared the performance of various pre-trained convolutional neural network (CNN) architectures, including VGG16, MobileNetV2, DenseNet121, and Vision Transformer (ViT), all pre-trained on the ImageNet dataset. The evaluation was conducted in two stages: (1) patch-based classification using annotated regions, and (2) WSI-based classification for whole slide images lacking specific region annotations.

### 5.1. Patch-Based Classifier Performance

**Figure 10:**
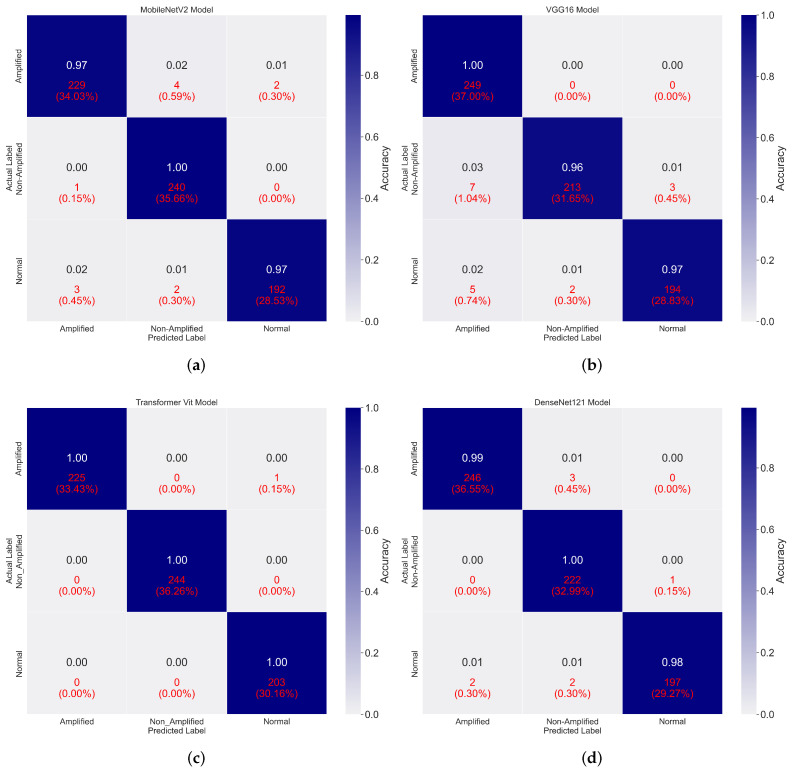
Confusion matrices illustrating the classification performance of the fine-tuned models: (**a**) MobileNetV2, (**b**) VGG16, (**c**) Vision Transformer (ViT), and (**d**) DenseNet121.

This section reviews the results of the patch-based classification performance of the deep-learning models. VGG16 achieved an accuracy of 97%, with precision, sensitivity, and F1-score of 98%, 97%, and 97%, respectively. DenseNet121 outperformed VGG16, with an accuracy of 99%, accompanied by precision, sensitivity, and F1-score of 99%, 98%, and 99%, respectively. MobileNetV2 performed comparably, with an accuracy of 98% and precision, sensitivity, and F1-score values of 98%, 99%, and 99%, respectively. The Vision Transformer (ViT) model achieved the highest performance, scoring 100% in accuracy, precision, sensitivity, and F1-score. Figure 10 shows the models’ confusion matrices, while Table 3 provides a detailed breakdown of the performance metrics for each class.

Error analysis highlighted that most misclassifications occurred in the Non-Amplified class, which was frequently confused with the Amplified class due to their overlapping staining characteristics. This suggests that future work should focus on advanced feature extraction methods to mitigate this issue. Additionally, some errors were attributed to image artifacts within the WSI, underscoring the importance of preprocessing steps.

**Figure 11:**
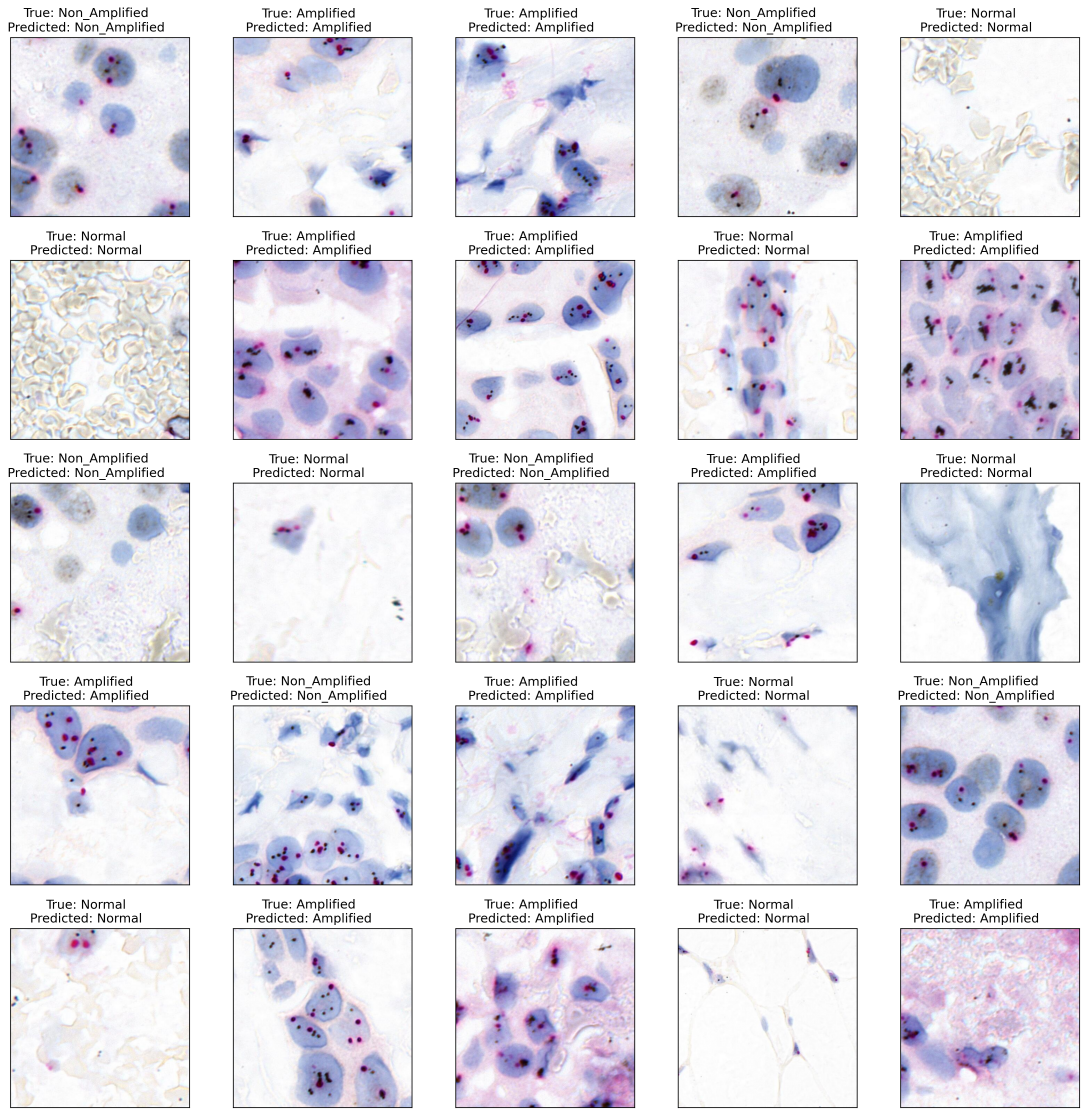
Examples of HER2-SISH patch samples with their respective model-predicted classifications.

Figure 11 displays annotated WSI regions based on patch classifier predictions, where red pseudo-regions denote Amplified areas identified by the deep-learning model. The model successfully recognized both stained and tumor regions while minimizing the misclassification of artifacts and Non-Amplified regions. Visual assessment and feedback from pathologists confirmed the model’s accuracy in identifying significant WSI regions, supporting its effectiveness.

### 5.2. WSI-Level Classifier Results

The term “WSI-level classifier” refers to the aggregated predictions derived from the patch-level classifier. Direct comparisons to other WSI-level classifiers are challenging due to the lack of WSI-level annotations.

In this section, we assess the Vision Transformer (ViT) model’s performance for classifying patches within whole slide images (WSIs). Following the patch-level evaluation, the top-performing ViT model was applied to classify WSI tiles. These patches were then combined to reconstruct the WSIs, with pseudo-color overlays indicating classification results: green for Normal, red for Amplified, and blue for Non-Amplified regions.

**Figure 12:**
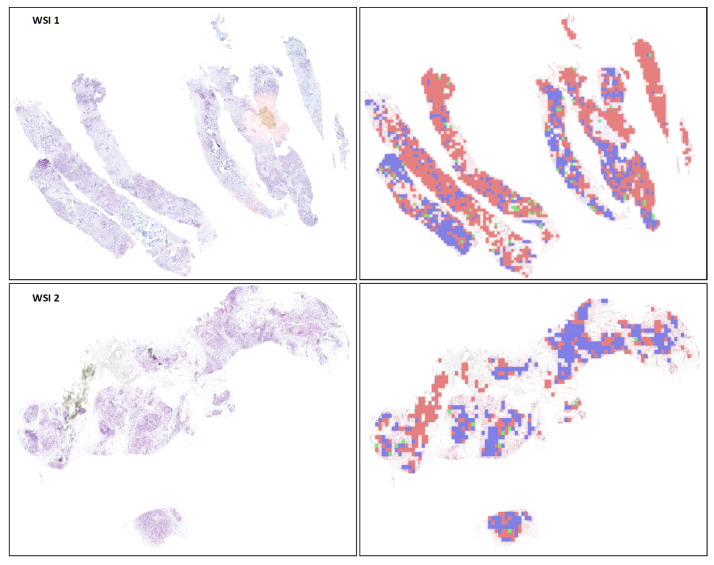
Visualization of classification results on WSIs with pseudo-color class maps. The figure illustrates the ViT model’s performance at the WSI patch level, with corresponding outputs shown on the right of each WSI.

Although direct WSI-level annotations were not available for comparative analysis, Figure 12 visually illustrates the classification results on WSIs with pseudo-color maps. Table 4 summarizes the model’s performance metrics on unseen data, showcasing its generalization capabilities. The use of generalizability concepts [47] ensures robustness, which is critical for medical image classification.

To further evaluate the model, expert-annotated ROIs were re-examined using the trained ViT model. The ViT model achieved an overall accuracy of 78% on these unseen regions. The detailed class-specific performance showed precision of 62%, sensitivity of 67%, and an F1-score of 62% across Normal, Non-Amplified, and Amplified categories.

**Figure 13:**
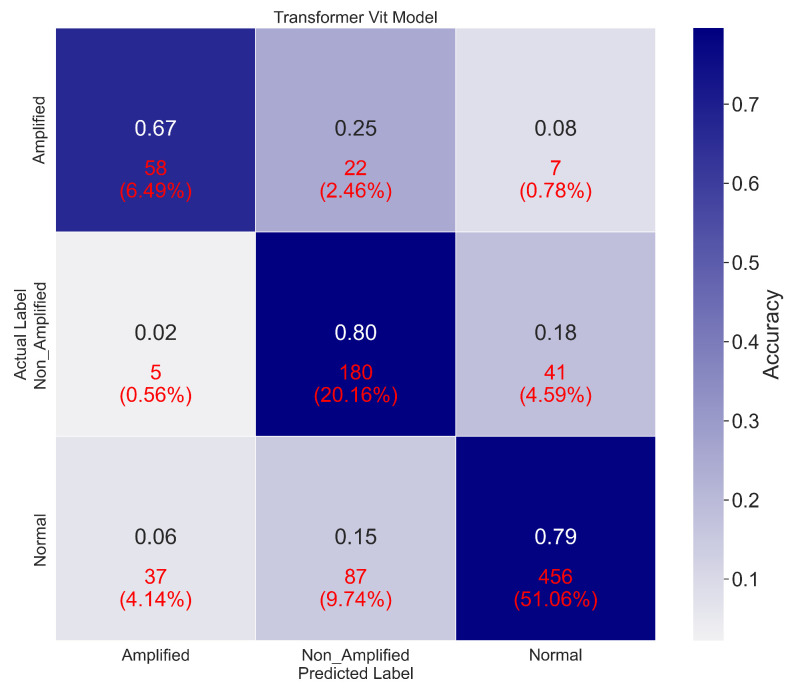
Confusion matrix illustrating the Vision Transformer (ViT) model’s performance on unseen test data, highlighting its generalization capabilities.

Figure 13 presents the confusion matrices for the ViT model’s performance on unseen WSI data. Table 4 provides a comparison of the quantitative performance of the ViT model and DenseNet121 on unseen data. While DenseNet121 reached 99% accuracy on training data, its performance on unseen data dropped to 52%, likely due to the presence of unprocessed white patches and outliers in real WSI data, which poses challenges for deep-learning models.

### 5.3. Analysis of Misclassified Patches

Misclassifications primarily occurred in the Non-Amplified class, frequently confused with Amplified due to similar staining intensities and uneven image quality. Artifacts, noise, and non-uniform staining were identified as contributing factors affecting feature extraction.

Key reasons for misclassification include the following:

- Staining Variability: Inconsistent staining across samples impacts classification accuracy.
- Artifacts and Noise: Background artifacts and uneven illumination disrupt model focus.
- Limited Data Diversity: Insufficient examples of complex patterns during training lead to reduced model generalization.

To enhance model performance:

- Stain Normalization: Standardize color variations to maintain focus on structural features.
- Advanced Attention Mechanisms: Use attention-based models to prioritize critical image regions.
- Artifact Removal: Employ preprocessing techniques for noise and artifact reduction.
- Data Augmentation: Increase training data variability to improve model robustness.

### 5.4. Discussion

This study presents an innovative approach for identifying Normal, Amplified, and Non-Amplified regions in HER2-SISH-stained whole slide images (WSIs). The proposed method automates the process of selecting regions from WSIs, generating class-specific predictions, overlaying pseudo-color maps, and reconstructing the WSI with these overlays. Unlike existing methods, which rely on manual region selection, such as those by Tewary et al. [48] and Saha et al. [49] that require manual patch generation from WSIs, our approach offers full automation. While methods like Mukundan et al. [50] and Singh and Mukundan [51] selected patches for HER2-IHC scoring manually, and others like Qaiser et al. [52] used attention mechanisms within pre-selected areas, our strategy focuses on automating background and artifact removal to enhance analysis accuracy and efficiency.

The system leverages a thresholding technique to filter out patches with over 50% white space, streamlining computational load and emphasizing tissue-rich regions. This significantly improves the reliability of HER2 scoring.

Most existing methodologies use patch generation at a magnification of 40×. Our study opted for a 20× magnification to balance computational resources and better align with pathologists’ typical viewing conditions. Comparing other HER2 scoring systems using different stains is challenging, but insights from systems like Her2Net by Saha et al. [49], which achieved 98.33% segmentation accuracy, serve as a qualitative reference.

**Figure 14:**
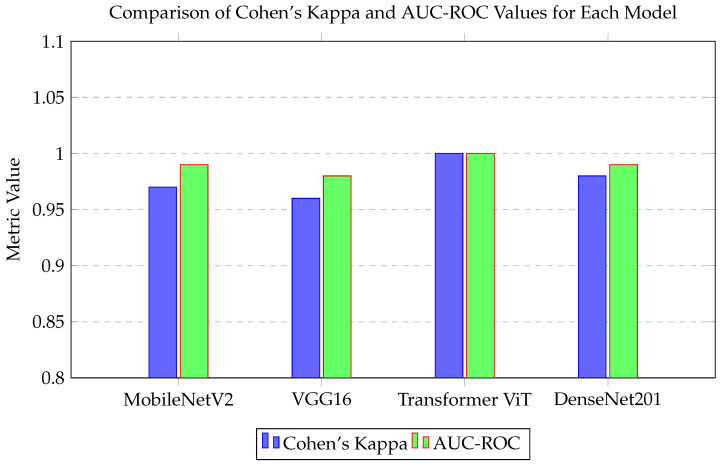
Comparison of Cohen’s Kappa and AUC-ROC values for each model.

We evaluated performance using Cohen’s Kappa and AUC-ROC for comprehensive model assessment. The ViT model achieved the highest Cohen’s Kappa value of 1.00 (Figure 14), indicating perfect alignment with true labels. The AUC-ROC also indicated superior discrimination ability, with ViT and DenseNet201 models achieving values close to 1.00. These metrics, along with Matthews Correlation Coefficient (MCC), underscore the models’ reliability, where ViT showed exceptional consistency.

### 5.5. Ablation Studies

We conducted a K-fold cross-validation (k=5) to confirm the robustness of the models, summarized in Table 5. The ViT model consistently performed well across all folds, while DenseNet201 demonstrated comparable reliability. MobileNetV2 and VGG16 also maintained strong performance metrics.

**Figure 15:**
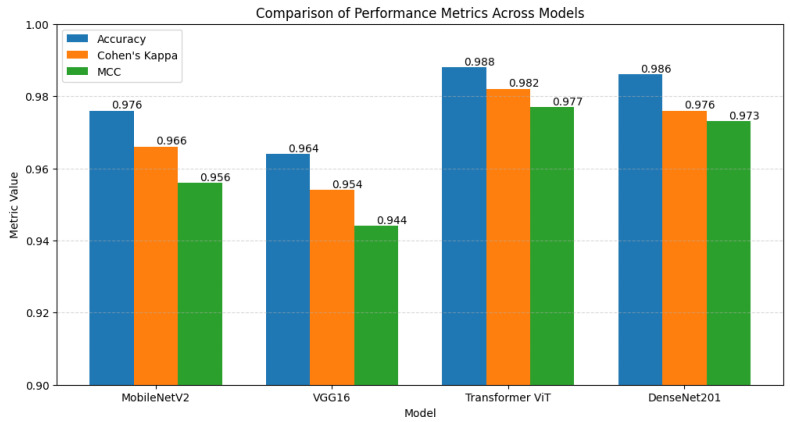
Comparison of performance metrics across models for accuracy, Cohen’s Kappa, and MCC.

Figure 15 visualizes model performance across different metrics for comparative insight.

## 6. Conclusions

This paper presents a comprehensive analysis of automated pipelines for classifying HER2-SISH-stained histopathology images into three categories: Normal, Amplified, and Non-Amplified. Our approach involves two primary stages: fine-tuning pre-trained deep learning models for patch-level classification and deploying the best-performing model for WSI region identification. This is the first study to report the classification of HER2-SISH images into these distinct classes and apply WSI-level region identification, enhancing HER2 scoring systems to aid breast cancer treatment.

The private clinical dataset used includes 46 HER2-SISH WSIs annotated by expert pathologists, with identified regions of interest (ROIs) for each class. These were preprocessed into uniform patches of size 512×512×3 for model training. Four models—MobileNetV2, VGG16, DenseNet121, and Vision Transformer (ViT)—were evaluated based on metrics such as precision, recall, F1-score, and accuracy. The ViT model outperformed the others, achieving 100% patch-level accuracy, and was applied for classifying WSI tiles, generating color-coded visualizations (green for Normal, red for Amplified, blue for Non-Amplified) to aid expert analysis.

Although direct quantitative WSI-level evaluation was limited due to the lack of ground truth annotations, the ViT model’s generalization was assessed with 78% accuracy on independent data, indicating robustness for WSI-level predictions.

Future research will focus on refining image preprocessing and incorporating stain normalization to enhance performance on unseen data. Additionally, we plan to deploy the proposed pipeline in real-time clinical environments and improve our web application to better assist medical professionals.

## Figures and Tables

**Table 1:** Statistical overview of images in the dataset obtained from the collaborating hospital. Note: Normal WSI is marked as NA because Normal regions were selected from Amplified (AMP) and Non-Amplified (Non-AMP) cases.

Sr. No.	Description	Amp	Non Amp	Normal
Dataset 1				
1	Number of WSIs per Class	20	17	NA
2	Number of ROIs	120	80	257
3	Number of Processsed Patches	1200	1206	956
Dataset 2				
1	Number of WSIs per Class	5	4	NA
2	Number of ROIs	25	20	42
3	Number of Patches	156	106	620

**Table 2:** Hyperparameters for the models.

Sr. No.	Model	Hyperparameter	Value
1		Input-Shape	224, 224, 3
		Include-top	FALSE
		Weights	imagenet
	MobileNetV2	Pooling	avg
		Output Activation	softmax
	VGG16	Dense Units	128
		Optimizer	adam
	DenseNet121	Loss	categorical_crossentropy
		Metrics	accuracy
		Batch Size	32
		Epochs	20
2	Transformer	Transformers_version	4.13.0.dev0
		Model_type	vit
		Input-Shape	224, 224, 3
		Hidden size	786
		Batch Size	32
		Num Attention Heads	12
		Num Hidden Layers	12
		Intermediate Size	3072
		Hidden Dropout Probability	0.1
		Attention Probs Dropout Probability	0.1

**Table 3:** Comprehensive performance metrics with balanced accuracy, MCC, and 95% confidence intervals. This table provides a detailed summary of classification performance on Dataset 1, showcasing metrics such as precision, sensitivity, F1-score, accuracy, balanced accuracy, and MCC, each with corresponding 95% confidence intervals. These results underscore the effectiveness and robustness of the evaluated models.

Techniques	Type of Class	Precision	Sensitivity	F1-Score	Accuracy	Balanced Accuracy	MCC
MobileNetV2	Amplified	0.97 ± 0.02	0.98 ± 0.01	0.98 ± 0.01	0.98 ± 0.01	0.99 ± 0.01	0.97 ± 0.01
	Non-Amplified	0.98 ± 0.01	1.00 ± 0.00	0.99 ± 0.01			
	Normal	0.99 ± 0.01	0.97 ± 0.01	0.98 ± 0.01			
VGG16	Amplified	0.98 ± 0.02	0.98 ± 0.01	0.98 ± 0.01	0.97 ± 0.01	0.98 ± 0.01	0.96 ± 0.01
	Non-Amplified	0.98 ± 0.01	0.97 ± 0.01	0.97 ± 0.01			
	Normal	0.96 ± 0.02	0.97 ± 0.01	0.97 ± 0.01			
Transformer ViT	Amplified	0.99 ± 0.01	1.00 ± 0.00	0.99 ± 0.01	0.99 ± 0.01	1.00 ± 0.00	1.00 ± 0.00
	Non-Amplified	0.99 ± 0.01	0.99 ± 0.01	0.99 ± 0.01			
	Normal	0.99 ± 0.01	0.98 ± 0.01	0.99 ± 0.01			
DenseNet201	Amplified	0.99 ± 0.01	1.00 ± 0.00	0.99 ± 0.01	0.99 ± 0.01	0.99 ± 0.01	0.98 ± 0.01
	Non-Amplified	0.99 ± 0.01	0.98 ± 0.01	0.99 ± 0.01			
	Normal	0.98 ± 0.01	0.98 ± 0.01	0.98 ± 0.01			

**Table 4:** Comprehensive performance metrics with balanced accuracy, MCC, and 95% confidence intervals. This table presents detailed quantitative results for the classification models evaluated on unseen data (Dataset 2). The metrics, including precision, sensitivity, F1-score, accuracy, balanced accuracy, and MCC with 95% confidence intervals, demonstrate the generalization performance and robustness of each model across different classes.

Techniques	Type of Class	Precision (95% CI)	Sensitivity (95% CI)	F1-Score (95% CI)	Accuracy (95% CI)	Balanced Accuracy (95% CI)	MCC (95% CI)
Transformer ViT	Amplified	0.58 (0.55, 0.61)	0.79 (0.76, 0.82)	0.70 (0.67, 0.73)	0.78 (0.76, 0.80)	0.78 (0.76, 0.80)	0.76 (0.74, 0.78)
	Non-Amplified	0.90 (0.88, 0.92)	0.80 (0.78, 0.82)	0.84 (0.82, 0.86)			
	Normal	0.62 (0.60, 0.64)	0.67 (0.65, 0.69)	0.62 (0.60, 0.64)			
DenseNet201	Amplified	0.18 (0.15, 0.21)	0.71 (0.68, 0.74)	0.28 (0.25, 0.31)	0.52 (0.50, 0.54)	0.52 (0.50, 0.54)	0.51 (0.49, 0.53)
	Non-Amplified	0.49 (0.47, 0.51)	0.48 (0.46, 0.50)	0.44 (0.42, 0.46)			
	Normal	0.89 (0.87, 0.91)	0.54 (0.52, 0.56)	0.67 (0.65, 0.69)			

**Table 5:** K-fold cross-validation results for performance metrics.

Model	Fold	Accuracy	Cohen’s Kappa	MCC
MobileNetV2	Fold 1	0.97	0.96	0.95
	Fold 2	0.98	0.97	0.96
	Fold 3	0.98	0.97	0.96
	Fold 4	0.97	0.96	0.95
	Fold 5	0.98	0.97	0.96
VGG16	Fold 1	0.96	0.95	0.94
	Fold 2	0.97	0.96	0.95
	Fold 3	0.96	0.95	0.94
	Fold 4	0.97	0.96	0.95
	Fold 5	0.96	0.95	0.94
Transformer ViT	Fold 1	0.99	0.98	0.98
	Fold 2	0.99	0.98	0.98
	Fold 3	0.99	0.99	0.98
	Fold 4	0.98	0.98	0.97
	Fold 5	0.99	0.98	0.98
DenseNet201	Fold 1	0.99	0.98	0.98
	Fold 2	0.99	0.98	0.98
	Fold 3	0.99	0.98	0.98
	Fold 4	0.98	0.98	0.97
	Fold 5	0.99	0.98	0.98

## Data Availability

The data used in this study were derived from human organ samples and are subject to privacy and ethical restrictions. Due to these restrictions, the dataset cannot be shared publicly. The use of this data has been approved by the hospital’s ethical review board for research purposes only. Access to the data can be granted to other researchers upon request, subject to signing appropriate ethical agreements to ensure privacy and confidentiality.
